# Uncoupling conformational states from activity in an allosteric enzyme

**DOI:** 10.1038/s41467-017-00224-0

**Published:** 2017-08-07

**Authors:** João P. Pisco, Cesira de Chiara, Kamila J. Pacholarz, Acely Garza-Garcia, Roksana W. Ogrodowicz, Philip A. Walker, Perdita E. Barran, Stephen J. Smerdon, Luiz Pedro S. de Carvalho

**Affiliations:** 1 Mycobacterial Metabolism and Antibiotic Research Laboratory, The Francis Crick Institute, 1 Midland Road, London, NW1 1AT UK; 2 Michael Barber Centre for Collaborative Mass Spectrometry, Manchester Institute of Biotechnology & School of Chemistry, University of Manchester, Manchester, M1 7DN UK; 3 Structural Biology Science Technology Platform, The Francis Crick Institute, 1 Midland Road, London, NW1 1AT UK; 4 Structural Biology of DNA-damage Signalling Laboratory, The Francis Crick Institute, 1 Midland Road, London, NW1 1AT UK

## Abstract

ATP-phosphoribosyltransferase (ATP-PRT) is a hexameric enzyme in conformational equilibrium between an open and seemingly active state and a closed and presumably inhibited form. The structure-function relationship of allosteric regulation in this system is still not fully understood. Here, we develop a screening strategy for modulators of ATP-PRT and identify 3-(2-thienyl)-l-alanine (TIH) as an allosteric activator of this enzyme. Kinetic analysis reveals co-occupancy of the allosteric sites by TIH and l-histidine. Crystallographic and native ion-mobility mass spectrometry data show that the TIH-bound activated form of the enzyme closely resembles the inhibited l-histidine-bound closed conformation, revealing the uncoupling between ATP-PRT open and closed conformations and its functional state. These findings suggest that dynamic processes are responsible for ATP-PRT allosteric regulation and that similar mechanisms might also be found in other enzymes bearing a ferredoxin-like allosteric domain.

## Abstract (web summary)

Active and inactive state ATP-phosphoribosyltransferases (ATP-PRTs) are believed to have different conformations. Here the authors show that in both states, ATP-PRT has a similar structural arrangement, suggesting that dynamic alterations are involved in ATP-PRT regulation by allosteric modulators.

## Introduction

Allostery is the change in protein activity triggered by binding of a ligand (effector) at a site different from the active site^1, 2^. Feedback allosteric circuits are a direct, rapid and efficient mechanism of metabolic control. These feedback allosteric circuits contribute to metabolic homeostasis by translating changes in the concentration of metabolites to enzyme activity and ultimately cellular responses^3^. Protein domains with a ferredoxin-like (FL) βαββαβ topology have been widely co-opted throughout evolution for allosteric control. Particular FL domains have evolved to recognize and respond to one -or sometimes more- molecules of very distinct nature, including nucleotides^4^, heavy metals^5^, and various amino acids. Aspartate carbamoyltransferase (an FL domain containing enzyme) is one of such enzymes, where multiple nucleotides inhibit or activate catalysis. Elegant work describing the complex analysis of regulation by multiple effectors (physiologic ligands) has been carried out^6^.

Feedback regulation via amino acid binding to FL domains is widespread in enzymes involved in amino acid biosynthetic pathways (Supplementary Fig. 1). Examples in the biosynthesis of aromatic amino acids include eukaryotic phenylalanine-4-hydroxylase (PAH; 1.14.16.1), regulated type Iβ 3-deoxy-D-*arabino*-heptulosonate 7-phosphate synthase (DAH7PS; 2.5.1.54)^7^ and the bacterial and plant chorismate mutase/prephenate dehydratase (PDT; 5.4.99.5/4.2.1.51)^8^. In branched amino acids biosynthesis, proteins regulated via FL domains are the bacterial and plant biosynthetic threonine ammonia-lyase (EC 4.2.1.19)^9^ and the bacterial acetohydroxyacid synthase isozyme III (AHAS; EC 2.2.1.6)^10^. Other examples are in Thr, Lys, Met and Ile biosynthesis, the bifunctional chloroplastic aspartokinase/homoserine dehydrogenase 2 (AKHSDH2; 2.7.2.4/ 1.1.1.3), and in Ser biosynthesis, D-3-phosphoglycerate dehydrogenase types I and II (PHGDH; 1.1.1.95). The long form of ATP-phosphoribosyltransferase (ATP-PRT; EC 2.4.2.17), the enzyme that catalyses the first committed step of l-histidine (l-His) biosynthesis, is also feedback inhibited by l-His^11, 12^ (Supplementary Figs. 2 and 3) via a FL domain. Many of these FL-regulated enzymes present in pathogenic bacteria, such as the ATP-PRT from *Mycobacterium tuberculosis*, are essential and have been proposed as attractive targets for antibacterial drug discovery.

Protein domain structure databases place the FL domain of ATP-PRT in the same superfamily as the cyclic-di-AMP receptor family, peptidase S54, and the GlnB proteins^13, 14^. The defining attribute of the GlnB superfamily is that the FL domains arrange in trimers with the β-sheet of each monomer positioned orthogonally to one another^14^. Because of the common FL fold and functional similarity, the regulatory domain of ATP-PRT has also been associated with the aspartate kinase, chorismate mutase and TyrA (ACT) superfamily^15^. The FL domains of GlnB and ACT have no known evolutionary relationships and represent a superfold that has likely emerged by convergence^16^.

Our understanding of the mechanisms of amino acid-binding FL domain-mediated allosteric inhibition comes mostly from the few available crystal structures of free vs. inhibited forms. For *T. maritima* DAH7PS and PHGDH, the available structures show that effector binding triggers domain reorganization leading to occlusion of the active site^17, 18^. In the case of ATP-PRT, an early study proposed that l-His binding caused a transition from dimer to hexamer^19^. However, more recent data show that both free and inhibited ATP-PRT are hexameric but that the l-His-bound form is more compact due to a slight rotation of the regulatory domains^12, 15, 20^. We now understand crystal structures as being the static snapshots of a continuum of conformations sampled by the enzyme. In this light, ATP-PRT can be described to exist in an equilibrium between a relaxed (R) hexamer and a tense (T) compact hexamer, with l-His binding preferentially to the R state and causing the population to shift to the T state^21, 22^.

**Fig. 1:**
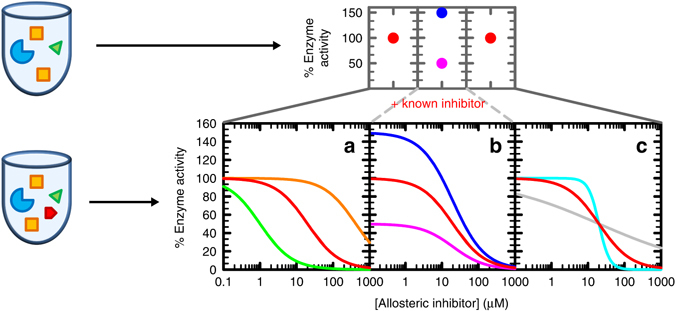
Schematic representation of CoSPI method vs conventional screenings. In the Compound Screening in the Presence of an Inhibitor (CoSPI) strategy, complete *IC*_50_ curves are recorded for a known inhibitor in the presence of each of the test compounds. The enzyme is in *blue*, substrates in *yellow*, known inhibitor in *red* and compound to test in *green*. The *top* panels show the single points observed in a conventional screening (*red dots* are missed hits, *blue* is an activator and *pink* an inhibitor). The *bottom* panels show the information-rich patterns observed using CoSPI. Curves depict simulated data for an inhibitor with *IC*_50_ = 20 µM, Hill number of 1 and capable of full inhibition (100 to 0% activity). The red curve are the data for the known inhibitor in the absence of other molecules. **a** Effects in the potency of the inhibitor. *Green*, increase in the apparent affinity of the inhibitor. *Orange*, decrease in the apparent affinity of the inhibitor. **b** Effects in the activity of the enzyme independently of the inhibitor. *Blue*, increase in the activity of the enzyme. *Pink*, decrease in the activity of the enzyme. **c** Effects in inhibitor-enzyme cooperativity. *Cyan*, increase in the cooperativity of inhibition. *Grey*, decrease in the cooperativity of inhibition

In an effort to better understand the allosteric regulation mechanism of ATP-PRT, we implemented a Compound Screening in the Presence of an Inhibitor (CoSPI) strategy (Fig. 1). CoSPI makes use of a known inhibitor to provide a targeted and detailed discovery of compounds that have an effect on activity of the enzyme as well as in its regulation. In contrast to most screens employed to date, CoSPI identifies molecules that are only active in the presence of a regulator-enzyme complex. This strategy led us to the discovery of 3-(2-Thienyl-l-alanine) (TIH) as a non-essential activator of *M. tuberculosis* ATP-PRT. Detailed kinetic measurements and structural characterization of ATP-PRT in the presence of TIH and/or l-His allowed us to probe the mechanism of allosteric regulation in ATP-PRT with unexpected results. Activation and inhibition of ATP-PRT leads to the same overall conformational tightening of the hexamer. This decoupling of activity and conformation indicates that allosteric regulation of ATP-PRT, and perhaps other FL domain containing allosteric enzymes, is more complex than the simple open–closed active–inactive model.

## Results

### CoSPI identifies TIH as an activator of ATP-PRT

**Fig. 2:**
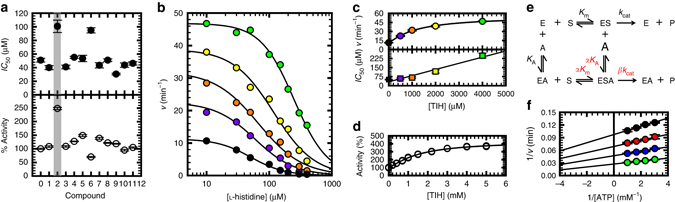
CoSPI discovery of a non-essential activator of ATP-PRT. **a** l-His *IC*_50_ and %Activity data obtained in the presence of each of 11 l-His analogues using CoSPI. The dashed lines mark l-His *IC*_50_ and the activity of uninhibited ATP-PRT. *Error bars* indicate the s.d. Number of replicates (*n*) = 3 **b** Representative inhibition curves determined for l-His in the presence of 0 (*black*), 0.5 (*purple*), 1 (*orange*), 2 (*yellow*) and 4 (*green*) mM compound 2, TIH (shown in the *upper right corner*). *Circles* are experimental measurements and *lines* the best fit to Eq. 2 in Methods section. **c** Replot of the data in **b**, showing hyperbolic increase in ATP-PRT activity (*circles*) and linear increase in l-His *IC*_50_ (*squares*), in the presence of TIH. The *lines* are the best fit to Eq. 1 in Methods and a linear regression of the data, respectively. *Error bars* indicate the s.e.m. **d** Steady-state kinetics of ATP-PRT activation by TIH. *Circles* are data points and the *line* is the best fit to Eq. 3 in Methods section, *n* = 2. **e** General modifier mechanism depicting an activator, E, enzyme; S, substrate; A, activator; P, product. **f** Double-reciprocal plot highlighting the non-essential activation pattern obtained when varying the concentration of TIH. *Circles* are data points obtained with 0 (*black*), 0.25 (*red*), 0.8 (*blue*) and 4 (*green*) mM TIH and *lines* are the best fit of the entire data set to Eq. 4 in Methods section. *Error bars* indicate the s.d., *n* = 2

We carried out a small, targeted version of CoSPI, to demonstrate its ability to uncover non-canonical regulators of enzyme function. By following the formation of phosphoribosyl-ATP (PR-ATP, *ε*_290_ = 3600 M^−1^ cm^−1^), we tested the effect of 11 commercially available l-His analogues, at a single concentration (1 mM), on the inhibition of ATP-PRT by l-His (Fig. 2a and Supplementary Fig. 4). Using as reference the activity values of uninhibited enzyme and the *IC*_50_ of l-His in the absence of any additional compounds (dashed lines in Fig. 2a), the selection of the best candidates was straightforward. Analogues 2 and 6 showed a change in l-His *IC*_50_ and uninhibited maximal activity. TIH, compound 2, was chosen for further characterization as it had the greatest effect. Addition of TIH to the reaction mixture resulted in a linear increase in the l-His *IC*_50_, and a hyperbolic (saturable) increase in the reaction velocity (Fig. 2b, c). We confirmed the activation of ATP-PRT by TIH by determining the concentration required to half-maximal activation (*AC*_50_) (Fig. 2d). TIH increases ATP-PRT activity up to 433 ± 18 %, with an *AC*_50_ of 1.5 ± 0.2 mM.

### TIH increases ATP-PRT turnover rate

According to the Botts and Morales general modifier mechanism (Fig. 2e)^23, 24^, the presence of a modifier (either an inhibitor or an activator) might have an effect on the dissociation constant of the ternary enzyme-substrate-modifier complex, this effect is given by the constant α. In addition, the modifier can have an effect on *k*_cat_, which is described by the coefficient β. Non-essential activation is generally characterized by a ligand that can bind and activate an enzyme, not being essential for enzymatic activity. Values of α lower than 1 and values of β higher than 1 are usually observed. However, mixed-type systems where α and β are higher than 1 are also possible, as are systems where α and β are both lower than 1^25^. We performed measurements of ATP-PRT activation by TIH at different concentrations of either of the substrates, ATP or PRPP. Coefficients α and β were consistent with non-essential activation in both cases (Fig. 2f, Supplementary Figs. 5 and 6). For ATP we obtained a *K*_A,ATP_ value of 1.5 ± 0.2 mM, an α_ATP_ of 1.4 ± 0.2 and a β_ATP_ value of 4.9 ± 0.2. The activity of the unactivated reaction was 0.17 ± 0.01 s^−1^ and the *K*_m,ATP_ 0.10 ± 0.01 mM, in close agreement with values previously reported^12^. In the case of varying concentrations of PRPP, *K*_A,PRPP_ was 2.1 ± 0.6 mM, α was 1.1 ± 0.4 and a β 4.9 ± 0.5. The activity of the unactivated reaction was 0.21 ± 0.01 s^−1^ and the *K*_m,PRPP_ 0.19 ± 0.03 mM. These results indicate that TIH has little effect on the stability of the tertiary complex, but is able to increase the catalytic rate by up to 490%.

### l-His and TIH binding are mutually affected

**Fig. 3:**
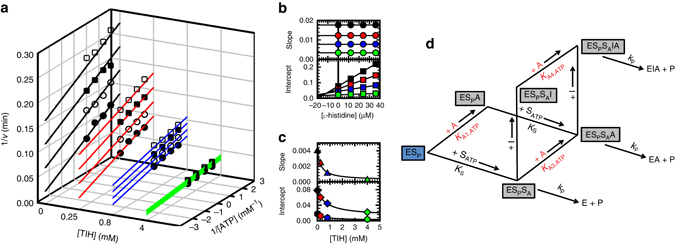
l-His inhibition kinetics in the presence of TIH. **a** Four double-reciprocal plots showing linear uncompetitive inhibition patterns determined for l-His in the presence of 0 (*black*), 0.25 (*red*), 0.8 (*blue*) and 4 (*green*) mM TIH, at fixed variable concentrations of ATP. Results are representative of two independent experiments. Points are experimental data obtained with 0 (*black circles*), 12 (*white circles*), 24 (*black squares*) and 36 (white squares) µM l-His. *Lines* are the best fit of each uncompetitive pattern data set to Eq. 5 in Methods section. **b** Secondary replot of the slopes (*circles*) and intercepts (*squares*) of the data shown in **a**. **c** Tertiary replots of the slopes (*top panel*) and intercepts (*lower panel*) of the data shown in **b**. Slope of the intercept (*triangles*), intercept of the intercept (*diamond*s) and intercept of the slope (*hexagons*). *Lines* are the best fit to Eq. 7 in Methods section. **d** Kinetic model for a combination of uncompetitive inhibition and non-essential activation. The complexes in *boxes* are in equilibrium. The *blue box* indicates that this complex is always present in the assay. E, Enzyme; S_A_, ATP; S_P_, PRPP; A, TIH; I, l-His; P, Product

We performed single inhibition measurements with co-variation of either substrate, ATP or PRPP, and l-His. Results showed linear uncompetitive inhibition by l-His vs. ATP, with a *K*_i,ATP_ of 20 ± 1.0 µM, and linear noncompetitive inhibition vs. PRPP, with a *K*_i,PRPP_ of 16 ± 1.0 µM as previously reported^12^. Following the method described by Andi and collaborators^26^, we repeated the inhibition experiment at four different concentrations of TIH (0, 0.25, 0.8 and 4 mM) (Fig. 3a). Increasing the concentration of TIH resulted in higher values of *V*_max_ and, therefore *k*_cat,_ and *K*_i,ATP_ for l-His inhibition (Table 1). As expected, the affinity of ATP was not considerably affected. A secondary replot of the data in Fig. 3a revealed a linear dependence of both the slopes and the intercepts as a function of l-His concentration (Fig. 3b). Tertiary replots of the slopes and intercepts of the data in Fig. 3b showed an exponential dependence on activator concentration (Fig. 3c). Three different *K*_A_ values could be determined from the tertiary replots using eq. 7 (Methods; Table 2). Each of the *K*_A_ values corresponds to the affinity of TIH to one of the following complexes, ES_P_ (intercept of the slope, *K*_A1,ATP_), ES_P_S_A_ (intercept of the intercept, *K*_A3,ATP_) and ES_P_S_A_I (slope of the intercept, *K*_A4,ATP_), where I is the inhibitor, S_A_ is ATP and S_P_ is PRPP, as illustrated in the model (Fig. 3d). The ratio of *K*_A3,ATP_/*K*_A4,ATP_ reports on the affinity of the activator for the ES_A_S_P_ complex, in the presence of the inhibitor, and is characterized by α_3/4,ATP_ = 2.60 ± 0.66; the value higher than 1 indicates that TIH has higher affinity for ATP-PRT in complex with l-His. Also determined from the tertiary replots (Supplementary Fig. 8a and Supplementary methods), the activation of ATP-PRT by TIH is higher when TIH binds to ATP-PRT in the presence of l-His. An 18-fold activation by TIH was observed in the presence of both substrates and l-His in contrast to a 6-fold activation in the absence of inhibitor; when TIH binds to ATP-PRT in complex with PRPP only a 11-fold activation is observed (Table 2 and Fig. 3d). When varying PRPP concentration (Supplementary Figs. 7 and 8b), four different *K*_A_’s could be determined. Once again, the results show that TIH has higher affinity for ATP-PRT in complex with l-His with values of α_1/2,PRPP_ = 1.92 ± 0.33 and α_3/4,PRPP_ = 3.27 ± 0.98, and fold-activation values were as high as 29, in the presence of l-His and ATP.

**Table 1:** Steady-state kinetic parameters for ATP-PRT inhibition in the presence of TIH

	[TIH] (mM)			
Parameters	0	0.25	0.8	4
*V*_max_ (s^−1^)	0.21 ± 0.01	0.28 ± 0.01	0.38 ± 0.01	0.76 ± 0.01
*K*_m,ATP_ (mM)	0.23 ± 0.03	0.20 ± 0.02	0.18 ± 0.02	0.16 ± 0.02
*K*_i,ATP_ (mM)	20 ± 1	25 ± 1	41 ± 2	89 ± 5

**Table 2:** Steady-state kinetic parameters for ATP-PRT activation in the presence of l-His

Parameters	Best fit	Fold activation
*K*_A1,ATP_ (mM)	0.50 ± 0.01	10.9
*K*_A2,ATP_ (mM)	—	—
*K*_A3,ATP_ (mM)	0.75 ± 0.10	6.6
*K*_A4,ATP_ (mM)	0.29 ± 0.06	18.1
α_1/2,ATP_	—	—
β_1/2 ATP_	2.60 ± 0.66	—

### TIH rescues ATP-PRT activity in the presence of l-His

**Fig. 4:**
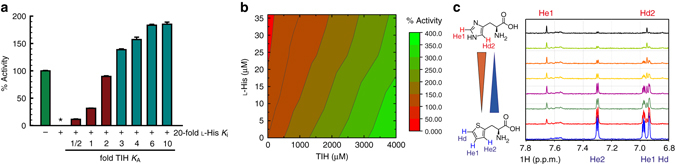
Competition between TIH and l-His. **a** ATP-PRT activity in the absence of l-His (*green bar*), in the presence of an l-His concentration equivalent to 20-fold *K*_i_ (0.4 mM; *star*) and in the presence of 0.4 mM l-His plus increasing concentrations of TIH (*dark red* and *cyan bars*, *K*_A_ = 2 mM). *Error bars* indicate the s.d., *n* = 3. **b** Contour plot of ATP-PRT activity landscape in the presence of both l-His and TIH. **c** STD-NMR of TIH and l-His with 20 μM ATP-PRT. TIH concentration was kept at 400 μM, while the concentration of l-His was varied, 0, 50, 100, 150, 400, 800, 1600 and 3000 μM, from the *blue bottom* trace, to the *black top* trace. Experiments were performed in 50 mM sodium phosphate buffer at pH 8.0. *Red* and n*avy blue* triangles indicate ATP-PRT occupancy by l-His and TIH, respectively

At a saturating concentration of l-His (400 µM, 20-fold *K*_i_), ATP-PRT is fully inhibited, showing no activity (Fig. 4a). Recovery of the activity can be achieved simply by addition of TIH to the inhibited reaction mixture. At a concentration close to 4 mM TIH (2-fold higher than *K*_A_ ~ 2 mM) 100% of the activity is restored and further activation is achieved with higher concentrations of TIH. Figure 4b shows a contour map that better illustrates the entire activity landscape obtained for ATP-PRT, in the presence of l-His and TIH, at all concentrations tested. Simultaneous binding of TIH and l-His to the hexamer was confirmed through a competition saturation transfer difference (STD) NMR experiment^27^. In an STD NMR experiment, signals are observed only for ligand protons in contact with the protein. In the absence of l-His signals of the TIH protons are observed (Fig. 4c, bottom spectrum). Increasing concentrations of l-His result in the progressive reduction of the TIH peaks and the appearance of l-His proton signals, indicating displacement of TIH by l-His.

### Both TIH and l-His-bound ATP-PRT are in the T conformation

We solved the crystal structures of ATP-PRT in complex with TIH or l-His by molecular replacement using the unliganded coordinates of the ATP-PRT ternary complex with l-His and AMP (1NH8) as a search model. Crystals of the two complexes grew in identical crystallization conditions and to comparable resolution, thus being an ideal case for a direct and detailed comparison between structures (Table 3). All ATP-PRT crystals obtained in complex with either l-His or TIH invariably belonged to the H 3 2 space group with one protein molecule in the asymmetric unit. The hexamer representing the biological assembly of ATP-PRT was obtained by symmetry operations preventing us from solving the structure of an ATP-PRT complex with both l-His and TIH.

**Table 3:** Data collection and refinement statistics (molecular replacement)

	ATP-PRT/TIH^a^	ATP-PRT/TIH	ATP-PRT/l-His
*Data collection*			
Space group	H 3 2 (155)	H 3 2 (155)	H 3 2 (155)
Cell dimensions			
*a*, *b*, *c* (Å)	115.48, 115.48, 124.60	117.51, 117.51, 127.36	117.01, 117.01, 127.33
α, β, γ (°)	90.00, 90.00, 120.00	90,00, 90.00, 120.00	90.00, 90.00, 120.00
Resolution (Å)	46.41–1.76	47.25–2.06	42.44–2.02
	(1.79–1.76)^b^	(2.12–2.06)^b^	(2.07–2.02)^b^
*R*_sym_ or *R*_merge_	0.033 (0.828)	0.041 (0.690)	0.033 (0.714)
*I*/σ*I*	20.6(1.0)	16.1(2.0)	22.3(2.1)
Completeness (%)	99.8 (98.2)	99.7 (99.9)	99.7 (100)
Redundancy	5.2 (3.6)	4.6 (4.8)	4.8 (5.1)
*Refinement*			
Resolution (Å)		47.25–2.06	42.44–2.02
		(2.12–2.06)	(2.07–2.02)
No. reflections		20989	22067
*R*_work_ / *R*_free_		0.1975/0.2307	0.1979/0.2378
No. atoms		2274	2284
Protein		2166	2161
Ligand/ion		26	36
Water		82	87
*B*-factors			
Protein		50.56	48.83
Ligand/ion		74.67	77.01
Water		52.47	52.90
R.m.s. deviations			
Bond lengths (Å)		0.004	0.007
Bond angles (°)		0.64	0.86

^a^Data set used for anomalous scattering difference map calculation

^b^Number of crystals was one for each structure. Values in parentheses are for highest-resolution shell

**Fig. 5:**
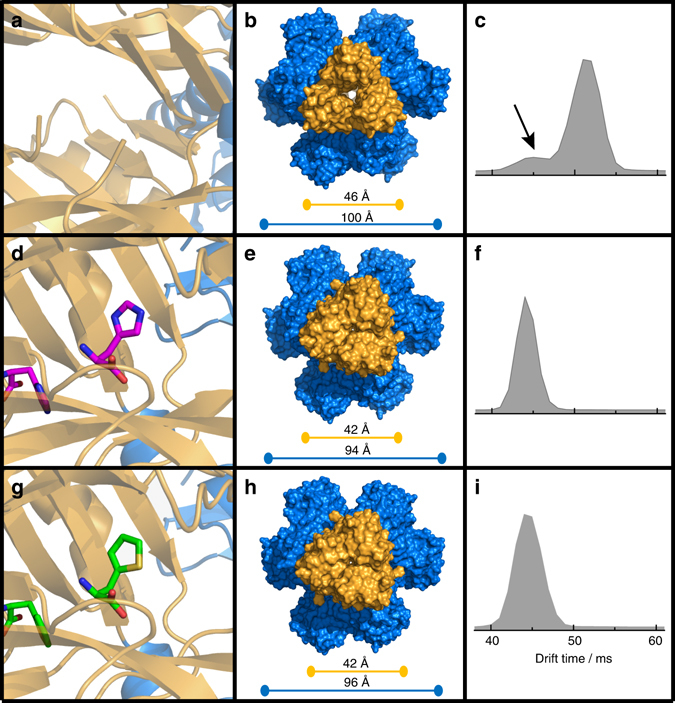
Crystal structure and native IM-MS of apo-, l-His-bound and TIH-bound ATP-PRT. Cartoon representation of the allosteric binding site in the hexamer, surface representation viewed from the top and native drift time distribution of: **a**, **b**, **c** Apo- (PDB code 1NH7); **d**, **e**, **f** l-His-bound (PDB code 5LHU), and **g**, **h**, **i** TIH-bound (PDB code 5LHT) ATP-PRT. Allosteric domains are coloured in *yellow* while the catalytic domains are coloured in *blue*. The *bars* represent the span of the different molecule complexes. Distances have been measured between coupled Cα atoms of I269 (*yellow bars*) and I129 (*blue bars*). The *black arrow* on **c** indicates the fraction of unliganded ATP-PRT in the T-state

**Fig. 6:**
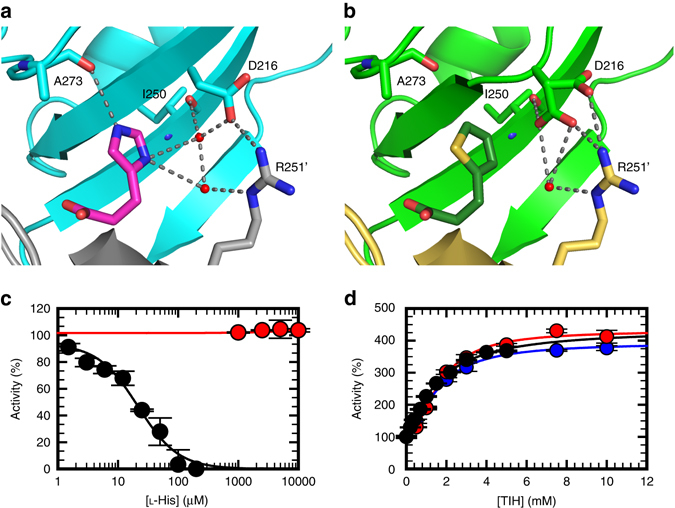
Differential conformations and kinetics of wild-type and D216 ATP-PRT in the presence of l-His or TIH. Cartoon representation highlighting the H-bonds surrounding l-His **a** and TIH **b** in their respective crystal structures. The D216 side chain in the TIH-bound ATP-PRT structure could be fitted in two conformations with 0.35 and 0.65 occupancy. Steady-state kinetics of ATP-PRT inhibition by l-His **c**, and activation by TIH **d**. WT (*black circles)*, D216 V (*red circles*) and D216 V + 2 mM l-His (*blue circles*), *error bars* indicate the s.d., n = 3. *Circles* are experimental measurements and *lines* are the best fit to Eq. 2 (Methods section) for inhibition by l-His and Eq. 4 (Methods section) for activation by TIH. Data for D216 V inhibition by l-His were fit to a linear regression. For WT, an *IC*_50_ for l-His of 22 ± 4 μM and an *AC*_50_ for TIH of 1.5 ± 0.2 mM were obtained. For D216 V, an *AC*_50_ for TIH of 1.7 ± 0.1 mM was obtained, which did not change in the presence of 2 mM l-His, with an *AC*_50_ of 1.5 ± 0.1 mM. l-His (up to 10 mM) did not change D216 V activity and therefore an *IC*_50_ could not be determined

As expected on the basis of the overall structural similarity between the two ligands and the results of the competition kinetics experiments, TIH was found to bind in the same allosteric site as l-His (Fig. 5a, d and g), a site defined by opposite and complementing surfaces of two adjacent regulatory FL-like domains. The position of the ligand in each complex was inferred by the presence of positive density in the unbiased difference map derived by molecular replacement (Supplementary Fig. 9). Fitting of the ligands and refinement of the structure resulted in identical ligand orientation (Fig. 5d and g). We confirmed the identity of TIH by calculating the anomalous scattering difference map for a data set of the complex collected at 1.8 Å, which exhibited positive density for the sulphur in the thiophenyl moiety and allowed unambiguous assignment of the correct orientation of the ring (Supplementary Fig. 10a). Side chains of the residues directly interacting with the ligand assume identical conformations in the two complexes with the only exception of D216 (Fig. 6a and b). In the ATP-PRT/l-His complex D216 interacts with the l-His side chain via a water molecule-mediated H-bond to ND1, but in the complex with TIH the water molecule is missing and its position occupied by one of two observed conformations for the D216 side chain which is oriented towards the thiophenyl ring. Taken together, the overall similarity between “inhibited” and “activated” structures and the differential conformation of D216 in both complexes could indicate that D216 is a “trigger” for inhibition of ATP-PRT. Steady-state kinetics studies on the D216V mutant support the interpretation of a specific role for D216 in l-His recognition and inhibition. D216V ATP-PRT is no longer inhibited by l-His (up to 10 mM); however, it is still activated by TIH, in the presence or absence of l-His, with similar affinity to the wild type (Fig. 6c and d). The other noticeable structural difference in the allosteric binding site is the absence in the TIH complex of the H-bond observed between HE2 of l-His and the carbonyl oxygen of A273. The missing H-bonds are likely to account for the three orders of magnitude difference in the apparent affinity of the two ligands and their opposite effect on enzymatic activity (Fig. 6).

The comparison between the structures of both the monomer and the crystallographic hexamer revealed that the l-His inhibited and the TIH activated complexes are highly superimposable and share the same tense (T) conformation so far attributed to the inactive state of the enzyme (Supplementary Fig. 11)^19, 20, 28^. Furthermore, no appreciable differences between the two complexes were observed in the side-chain orientation of the residues expected to be involved in substrate and/or product binding by virtue of similarity with the *E. coli* ATP-PRT in complex with PR-ATP (PDB code: 1Q1K)^28^ and *C. jejuni* ATP-PRT in complex with ATP (PDB code: 4YB7)^20^ (Supplementary Fig. 12). The mean global RMSD between the two complexes calculated over the entire sequence (residues 1–284) was 0.12 and 0.61 Å for the backbone and heavy atoms, respectively. The RMSD value between the open unliganded ATP-PRT (1NH7) and the structures in this work was of 4.24/4.56 Å (backbone/heavy atoms) and 4.24/4.55 Å for the l-His- and TIH-complexes, respectively. The crystallographic structures at 1.8 Å of the ATP-PRT/TIH complex obtained from different crystallization conditions consistently showed the hexamer in a T-state (Supplementary Fig. 10b).

### Native mass spectrometry supports R to T conformation shift

To complement our crystallographic results we employed native ion-mobility mass spectrometry (IM-MS) to investigate the equilibrium between the R- and T-states in the presence of either l-His or TIH. We obtained data for unligated ATP-PRT and in complex with l-His or TIH, at neutral pH (6.8) and in mildly alkaline pH (9.0) conditions in which ATP-PRT is more active^12^ (Supplementary Table 1). Results at neutral pH (Fig. 5) showed the existence of a pre-existing equilibrium between the R- (drift time of 51.2 ms) and T- (drift time of 44.0 ms) states, with a strong preference for the R-state. Addition of either l-His or TIH shifted the equilibrium towards shorter drift times (44.1 and 44.2 ms, respectively), characteristic of the T-state. At basic pH, unligated, l-His and TIH-bound ATP-PRT are all in the T-state (Supplementary Fig. 13).

## Discussion

Target-based small-molecule screenings are a valuable and widely used approach for lead generation in drug discovery. In traditional screenings compounds are tested against purified enzymes, with the goal of detecting enzyme inhibition^29, 30^, activation^31^, or binding to the target protein^32^, while other screenings test for secondary ligands that only bind after the first ligand binds^33, 34^. Despite their undisputed usefulness, traditional screens fail to detect compounds displaying less common or peculiar effects, such as influencing the efficacy or affinity of a known effector, or altering effector–enzyme cooperativity. In order to screen for these effects, we developed CoSPI, a targeted screen of small molecules. The screen is carried out in the presence of a known inhibitor, and consists of determining and evaluating the entire inhibition profile (*IC*_50_ curve) of the known inhibitor in the presence of a single fixed concentration of each of the compounds. Hill number (*n* ^H^), *V*_max_ and *IC*_50_ are then estimated from such curves and compared. Three limiting behaviours can potentially be observed using a CoSPI design (Fig. 1). First, compounds that have no effect on their own but are able to increase or decrease the potency of the known inhibitor (Fig. 1a). Second, compounds able to alter the enzyme activity independently of the known inhibitor (Fig. 1b). And third, compounds that do not alter enzyme activity but alter the inhibitor-enzyme cooperativity (Fig. 1c). As proof of concept, we used the CoSPI strategy to screen 11 l-His analogues for their ability to modify *M. tuberculosis* ATP-PRT activity in the presence of its allosteric inhibitor l-His. The outcome of the screen was simple and clear (Fig. 2a), and lead to the discovery of TIH, the first reported allosteric activator of ATP-PRT and in the whole histidine biosynthesis pathway. Allosteric activators have only recently emerged as valuable tools for the study of conformational changes associated with enzyme activation, and of the biological consequences of enzyme activation in the context of a specific signalling/metabolic pathway^35^.

TIH is a non-essential ATP-PRT activator of weak molar potency with *K*_A,ATP_ value of 1.5 ± 0.2 mM and *K*_A,PRPP_ value of 2.1 ± 0.6 mM. Nonetheless, it is able to activate ATP-PRT by up to 500%. TIH activation originates solely from a *V*-type effect, this is, it increases the catalytic rate (β-value of 4.9) but has no effect on *K*_m_ (α-value ~1); this is a different type of activation from most cases reported to date^36–39^. To the best of our knowledge, a fivefold increase in *k*_cat_ is one of the highest increases reported for a small-molecule allosteric activator. Having a similar structure and judging by its effect on l-His *IC*_50_ (Fig. 2b and c) it was expected that TIH and L-His bound at the same site. The results of the inhibition studies with co-variation of either of the substrates and L-His, at four different concentrations of TIH (Fig. 3a and Supplementary Fig. 7a) further supported this idea. In these experiments, increasing concentrations of TIH did not significantly affect the affinity of the substrates, but *K*_i_ values for L-His increased linearly, indicating a strong effect of TIH on l-His potency (Table 1 and Supplementary Fig. 7b). Furthermore, and despite its weak potency, TIH at a concentration of twice *K*_A_ (4 mM) was sufficient to fully activate ATP-PRT in the presence of l-His at a concentration ≈ twice *K*_i_ (36 μM). Under these conditions *V*_max_ reached values close to those obtained in the presence of saturating concentration of TIH (absence of l-His), *V*_a,max_, justifying the increase in fold-activation values from 6 to 18-fold. To confirm this behaviour, ATP-PRT activity was tested at increasing concentrations of TIH in the presence of l-His at a concentration of 20 times *K*_i_ (Fig. 4a), which fully abolishes activity. Again we saw that TIH at a concentration of 2 times *K*_A_ is sufficient to rescue ATP-PRT to normal activity levels. This apparently counterintuitive behaviour, indicative of a “kinetic superiority” of a low affinity ligand over a high affinity ligand may occur because there are six l-His binding sites in the ATP-PRT hexamer^12, 19^. The *n* ^H^ for l-His inhibition is 1.5^12^, suggesting that occupation of four of the six binding sites is sufficient for full inhibition of the hexamer, leaving two sites available for TIH to bind and activate ATP-PRT (Supplementary Fig. 14). In agreement with this hypothesis, TIH affinity is higher when l-His is bound to ATP-PRT, as revealed by values of α_3/4,ATP_, α_1/2,PRPP_ and α_3/4,PRPP_ of 1.9-, and 3.3- and 2.6-fold, respectively. This preference of TIH for the ATP-PRT- l-His complex is substrate independent. In addition, the fold-activation obtained in the presence of l-His is considerably higher than the fold-activation obtained in its absence (Table 2 and Supplementary Fig. 7e), this is because the very low *V*_max_ values observed in the presence of l-His increase substantially upon addition of TIH, until reaching *V*_a,max_, giving higher values of fold-activation. There have been a few reports of activators of bacterial enzymes in the literature, including activators of cylindrical proteases^40, 41^, proteasome-related HslV peptidase^42^ and glycoside hydrolase^39^. Nonetheless, TIH seems to be a distinct small-molecule activator of a bacterial enzyme, binding in the same site as the natural allosteric inhibitor.

The similarity between the crystallographic structures of the ATP-PRT hexamer in complex with the l-His or with TIH was surprising. Despite exerting an opposite effect on the enzymatic activity, both induce a rigid body conformational change that re-orients the regulatory domain with respect to the catalytic region leading to the compaction of the hexamer characteristic of the T-state. To rule out the possibility that this was a crystallization artefact, we performed measurements in the “vapour phase” using native IM-MS, with equivalent results. Our data suggest that there is not direct correlation between R- and T-states and active and inactive conformations in ATP-PRT. Instead, it is likely that other conformational states, less populated than R- and T-, are critical for activity and/or that there is an important dynamic contribution to the allosteric mechanism.

Given that substrate binding and product release can only take place in the R state, the R- and T-states might reflect the functional catalytic ‘cycle’ of ATP-PRT. We hypothesize that activators and inhibitors have opposite effects on the rate of sampling of the conformational ensemble. Thus, an activator may increase turnover by accelerating the sampling rate, while an inhibitor might slow it down. This hypothesis is consistent with the fact that TIH activation is solely due to an increase in *V*_max_. A detailed characterization of the conformational equilibrium exchange rates in the presence and absence of effectors will be required to test this hypothesis and is currently in progress. Of particular interest is the possibility that the uncoupling between R- and T-states and activity observed for ATP-PRT might be a feature of other ferredoxin-like domain-containing allosteric enzymes.

## Methods

### Materials

Buffers and l-His were purchased from Fisher Scientific. Except if specified otherwise, all other chemicals were purchased from Sigma-Aldrich. Ni-NTA resin and BL21(DE3)pLysS cells were purchased from Millipore. Complete EDTA-Free protease inhibitor was purchased from Roche.

### Protein purification

All steps of ATP-PRT and PPase purification were performed at 4 ˚C using an AKTA purifier 10 (GE Healthcare). Frozen BL21(DE3)pLysS (*pJ411::hisG* and *pJ411::ppa*) 50 grams of cells were thawed on ice, resuspended in buffer A (20 mM triethanolamine (TEA) pH 7.8, 300 mM NaCl, and 50 mM imidazole containing lysozyme, Complete® protease inhibitor cocktail and DNase) and lysed by sonication. After centrifugation at 25000 *g* for 30 min, the soluble fraction was loaded on a 100 ml Ni-NTA column and protein eluted by a 0 to 100% gradient of buffer B (20 mM TEA pH 7.8, 300 mM NaCl and 500 mM imidazole). UV-Vis peak fractions were analysed by SDS-PAGE, performed on a PhastSystem (GE Healthcare). Fractions containing only ATP-PRT or PPase were pooled, dialyzed into 20 mM TEA (pH 7.8), concentrated, flash-frozen and stored at −80 ˚C. This protocol allowed the purification to homogeneity of 1 g of ATP-PRT and 0.5 g of PPase. ATP-PRT concentration was determined using absorbance at 280 nm (*ε*_280_ = 20,800 M^−1^ cm^−1^). After the Ni-NTA column step, ATP-PRT protein for crystallization was further purified by gel filtration using a Sephadex S200 16/600 column and concentrated in the elution buffer (20 mM Tris-HCl pH 7.8, 100 mM NaCl) to a final concentration of 13 mg/ml before flash freezing.

### ATP-PRT activity measurements

Steady-state kinetic measurements were conducted at 25 °C ± 0.2 °C with a Shimadzu UV-2550 spectrophotometer equipped with dual-beam optics and a Peltier system for temperature control. Initial velocities for the forward reaction of ATP-PRT were measured by following the formation of PR-ATP (*ε*_290_ = 3,600 M^−1^ cm^−1^), in the presence of PPase. Pyrophosphatase is essential for this assay, as the equilibrium constant lies towards formation of ATP and PRPP. A typical reaction mixture contained 50 mM Tris-HCl (pH 8.5), 7 mM MgCl_2_, 200 mM KCl, 3 mM ATP, 1.5 mM PRPP, 600 nM PPase and 450 nM ATP-PRT.

### l-His analogue screening

To determine the effect of 11 commercially available analogues on l-His, ATP-PRT activity was assayed in the presence of saturating concentrations of substrates and metals, variable concentrations of l-His and in the presence of 1 mM of each of the analogues (with the exception of compound 1, where a concentration of 0.9 mM was used). To determine the effect of each analogue on enzyme activity (in the absence of l-His), ATP-PRT activity was measured in the presence of saturating concentrations of substrates and metals, and variable concentrations of each analogue (up to 5 mM).

### Activation kinetics

To determine the steady-state activation parameters and patterns associated with TIH, ATP-PRT activity was studied in the presence of variable concentrations of one substrate, PRPP or ATP, fixed saturating concentrations of the co-substrate (ATP or PRPP) and metals, and several fixed concentrations of TIH.

### Inhibition kinetics in the presence of activator

To determine the interaction between l-His and TIH, ATP-PRT activity was studied in the presence of variable concentrations of one substrate, PRPP or ATP, fixed saturating concentrations of the other substrate (ATP or PRPP) and metals, and several fixed concentrations of l-His. The experiment was repeated at several fixed concentrations of TIH.

### Activity rescue by TIH

ATP-PRT activity was studied in the presence of 0.4 mM l-His, a concentration 20-fold higher than l-His *K*_i_, which fully inhibited ATP-PRT. Increasing concentrations of TIH were then added to the reaction, up to 8 mM TIH, a concentration 4-fold higher than TIH *K*_A_.

### ^1^H NMR spectroscopy

NMR spectra were recorded at 25 °C on a Bruker Avance-600 spectrometer equipped with a triple resonance cryo-probe and operating at a ^1^H frequency of 600 MHz. Saturation transfer difference (STD) spectra^43^ of 20 µM ATP-PRT in the presence of fixed concentration of TIH and variable concentrations of l-His were recorded in 50 mM sodium phosphate buffer (pH 8.0). Spectra with on-resonance pre-saturation of the enzyme at 0.8 ppm were recorded with a saturation time of 2 s. An off-resonance control spectrum was recorded with a saturation time of 2 s. Water suppression was achieved by excitation sculpting^44^. Data processing and spectral subtractions were performed using Bruker TopSpin 3.2 software.

### Crystallization and X-ray data collection

All crystals of ATP-PRT complexes with either l-His or TIH were obtained by co-crystallization experiments at 18 °C using the sitting drop vapour diffusion method. Drops of 0.2 µl (protein stock:well solution 1:1) were set-up with the assistance of the Oryx protein crystallization robot. Protein concentration was 9.3 mg/ml whereas the ligands concentration was 100 µM and 5.5 mM for l-His and TIH, respectively. Crystals of ATP-PRT complexes with TIH and l-His were obtained from condition 4 of Classics Lite Suite crystallization screen by Qiagen (2.5 %(v/v) isopropanol, 1.0 M ammonium sulphate). Crystals of ATP-PRT in complex with TIH grown in the presence of ATP, although no positive density for ATP molecule could be located, were obtained from condition 71 of the Protein Complex Suite by Qiagen (0.1 M Sodium acetate anhydrous pH 5.0, 1.5 M Ammonium sulphate). Crystals were frozen adding 20 % (v/v) glycerol to the mother liquor for cryo-protection and stored in liquid nitrogen until data collection. Diffraction data were collected at beamline IO4 at the Diamond Light Source, UK. Data collection temperature was 100 K. Wavelength for both ATP-PRT/TIH and ATP/PRT/l-His datasets was 0.9282. Integration and scaling were performed using XDS and XSCALE via *xia2* expert system for X-ray diffraction data processing^45, 46^. Crystals belong to the rhombohedral space group H 3 2 with one molecule in the crystallographic asymmetric unit, which gives a solvent content of 54–56%. Subsequent data handling was carried out using the CCP4 software package^47^.

### Structure determination and refinement

Molecular replacement was carried out with PHASER^42^, using as a search model the coordinates of *M. tuberculosis* ATP-PRT complexed with l-His and AMP (PDB code 1NH8), but with the ligands and solvent molecules removed. Models were improved and refined alternating cycles of model correction by Coot^43^ and restrained refinement by Refmac^44^ and Phenix.Refine^45^. Figures were drawn using either Coot^43^ or PyMol^46^. For each structure, 284 amino acids of the native protein are visible in the molecule. Residues R26 to V40 and R184 to E194 could be fitted, but display higher flexibility as evidenced by less well-defined electron density. The bound ligands are clearly defined in both structures and refine well. A summary of the data collection and refinement statistics is given in Table 3. Ramachandran statistics are 97, 3.2 and 0.0 % for most favoured, additionally allowed and disallowed residues of ATP-PRT/TIH and 98, 1.4 and 1.1% for ATP-PRT/l-His.

### Native ion-mobility mass spectrometry

On the day of analysis, the buffer was exchanged to 100 mM ammonium acetate buffer (Fisher Scientific, Loughborough, UK) of specified pH, using micro Bio-Spin Chromatography columns (Micro Bio-Spin 6 Columns, Tris) following the instructions specified by the manufacturer. The desalting procedure was performed four to five times to achieve desired sample quality. pH of the buffer was adjusted with ammonia supplied by VWR International Ltd (UK). Solution pH readings were taken using a pH metre (Jenway 3305). High purity water was obtained from an Arium 611 water purification unit (Sartorius, Göttingen, Germany) fitted with a 0.2 µm filter.

The IM-MS data were acquired on a Synapt G2S HDMS (Waters, Manchester, UK). Samples were ionized using nano-elecrospray ionization (nESI) method, where protein species were charged and transferred from solution into the gas phase. nESI capillaries were prepared in-house from thin-walled borosilicate capillaries (inner diameter 0.9 mm, outer diameter 1.2 mm, World Precision Instruments, Stevenage, UK) using a Flaming/Brown P-97 micropipette puller (Sutter Instrument Company, Novato, CA, USA). These were then filled with sample using micro-loading tips (Eppendorf, Hamburg, Germany). A positive voltage was applied to the solution via 0.125 mm platinum wire (Goodfellow Cambridge Ltd., Huntingdon, UK) inserted into the capillary. Gentle conditions were applied to preserve the native-like structure: capillary voltage 1.6 kV, sampling cone 99 V, source temperature 20 °C, trap collision energy 5.6 V, and transfer collision energy 2 V. The pressure of the backing region was 8.3 mbar. For IM-MS, the helium cell and the IMS gas flows were 180 and 90 ml min^−1^, respectively, the IMS wave velocity was 617 m s^−1^, and the IMS wave height was 40 V. Nitrogen was the carrier gas. Data were acquired and processed with MassLynx software (Waters, Manchester, UK).

### Steady-state data analysis

Steady-state data were fitted using the nonlinear, least-squares, curve-fitting programs of Sigma-Plot for Windows, version 11.0. Individual saturation curves were fit to Eq. 1

1 $$v = VS{\rm{/}}\left( {S + K} \right)$$

where *V* is the maximal velocity, *S* is the substrate concentration, and *K* is the Michaelis constant for the substrate (*K*_m_). Inhibition data obtained under saturating concentrations of substrates and metals, and variable concentration of l-His, were fit to Eq. 2

2 $$v = {v_0}{\rm{/}}\left[ {1 + {{\left( {I{\rm{/}}I{C_{50}}} \right)}^{{n_{\rm{H}}}}}} \right]$$

where *v*_0_ is the uninhibited velocity, *I* is the l-His concentration, *IC*_50_ is the concentration of l-His necessary to give 50% inhibition and *n*_H_ is the Hill number. Activation data obtained under saturating concentrations of substrates and metals and variable concentration of TIH were fit to Eq. 3 ^48, 49^

3 $$v = {v_0} + \left\{ {\left[ {({v_{{\rm{amax}}}} - {v_0}){\rm{ \times }}{A^{{n_{\rm{H}}}}}} \right]{\rm{/}}\left[ {AC_{50}^{{n_{\rm{H}}}} + {A^{{n_{\rm{H}}}}}} \right]} \right\}$$

where *v*_amax_ is the maximal velocity at maximum activation, *A* is the concentration of the activator TIH and *AC*_50_ is the concentration of TIH necessary to increase the velocity by 50%. Activation data showing linear, non-essential activation pattern in a double-reciprocal plot were fit to Eq. 4 ^38, 39^

4 $$v = VS{\rm{/}}\left\{ {K\left[ {\left( {1 + A{\rm{/}}{K_{\rm{A}}}} \right){\rm{/}}\left( {1 + \beta A{\rm{/}}\alpha {K_{\rm{A}}}} \right)} \right]} {\rm{ + }}S\left[ {\left( {1 + A/\alpha {K_{\rm{A}}}} \right){\rm{/}}\left( {1 + \beta A{\rm{/}}\alpha {K_{\rm{A}}}} \right)} \right]\right\}$$

where *K*_A_ is the activator dissociation constant, *α* is the constant that denotes the modification of the substrate and activator dissociation constants in the presence of each other and *β* is the constant of the modification of the catalytic constant. Inhibition data showing linear, uncompetitive patterns in double-reciprocal plots were fit to Eq. 5

5 $$v = VS{\rm{/}}\left[ {K + S\left( {1 + I/{K_{\rm{i}}}} \right)} \right]$$

where *K*_i_ is the dissociation constant for the enzyme-inhibitor complex. Inhibition data showing linear, noncompetitive patterns in double-reciprocal plots were fit to Eq. 6

6 $$v = VS/\left[ {K\left( {1 + I{\rm{/}}{K_{\rm{i}}}} \right) + S\left( {1 + I{\rm{/}}{K_i}} \right)} \right]$$

Tertiary replots of the slopes and intercepts against activator concentration were fit to Eq. 7 ^50^

7 $${\rm{Slope}}/{\rm{Intercept}} = \left[ {{y_0} + {y_\infty }\left( {A{\rm{/}}{K_{\rm{A}}}} \right)} \right]{\rm{/}}\left[ {1 + \left( {A/{K_{\rm{A}}}} \right)} \right]$$

where *y*_0_ is the value of slope or intercept in the absence of activator and *y*_*∞*_ is the value of slope or intercept in the presence of infinite concentration of activator.

### Data availability

The atomic coordinates and the structure factors for *M. tuberculosis* ATP-PRT in complex with l-His and TIH have been deposited into the Protein Data Bank under the accession codes 5LHU and 5LHT. The UniProt accession codes P9WMN1 and P9WI55 for *M. tuberculosis* ATP-PRT and PPiase, respectively were used in this study. All other data are available from the corresponding author on reasonable request.

## Electronic supplementary material

Supplementary Information

## References

[1] Goodey NM, Benkovic SJ (2008). Allosteric regulation and catalysis emerge via a common route. Nat. Chem. Biol..

[2] Nussinov R, Tsai CJ (2013). Allostery in disease and in drug discovery. Cell.

[3] Lindsley JE, Rutter J (2006). Whence cometh the allosterome?. Proc. Natl Acad. Sci. USA.

[4] Wild JR, Loughrey-Chen SJ, Corder TS (1989). In the presence of CTP, UTP becomes an allosteric inhibitor of aspartate transcarbamoylase. Proc. Natl Acad. Sci. USA.

[5] Smith AT, Smith KP, Rosenzweig AC (2014). Diversity of the metal-transporting P1B-type ATPases. J. Biol. Inorg. Chem..

[6] Rabinowitz JD (2008). Dissecting enzyme regulation by multiple allosteric effectors: nucleotide regulation of aspartate transcarbamoylase. Biochemistry.

[7] Shumilin IA, Bauerle R, Wu J, Woodard RW, Kretsinger RH (2004). Crystal structure of the reaction complex of 3-deoxy-D-arabino-heptulosonate-7-phosphate synthase from Thermotoga maritima refines the catalytic mechanism and indicates a new mechanism of allosteric regulation. J. Mol. Biol..

[8] Tan K (2008). Structures of open (R) and close (T) states of prephenate dehydratase (PDT)--implication of allosteric regulation by L-phenylalanine. J. Struct. Biol..

[9] Gallagher DT (1998). Structure and control of pyridoxal phosphate dependent allosteric threonine deaminase. Structure..

[10] Kaplun A (2006). Structure of the regulatory subunit of acetohydroxyacid synthase isozyme III from Escherichia coli. J. Mol. Biol..

[11] Ames BN, Martin RG, Garry BJ (1961). The first step of histidine biosynthesis. J. Biol. Chem..

[12] Pedreno S, Pisco JP, Larrouy-Maumus G, Kelly G, de Carvalho LP (2012). Mechanism of feedback allosteric inhibition of ATP phosphoribosyltransferase. Biochemistry.

[13] Sillitoe I (2015). CATH: comprehensive structural and functional annotations for genome sequences. Nucleic. Acids. Res..

[14] Murzin AG, Brenner SE, Hubbard T, Chothia C (1995). SCOP: a structural classification of proteins database for the investigation of sequences and structures. J. Mol. Biol..

[15] Lang EJ, Cross PJ, Mittelstadt G, Jameson GB, Parker EJ (2014). Allosteric ACTion: the varied ACT domains regulating enzymes of amino-acid metabolism. Curr. Opin. Struct. Biol..

[16] Orengo CA, Jones DT, Thornton JM (1994). Protein superfamilies and domain superfolds. Nature.

[17] Cross PJ, Dobson RC, Patchett ML, Parker EJ (2011). Tyrosine latching of a regulatory gate affords allosteric control of aromatic amino acid biosynthesis. J. Biol. Chem..

[18] Thompson JR, Bell JK, Bratt J, Grant GA, Banaszak LJ (2005). Vmax regulation through domain and subunit changes. The active form of phosphoglycerate dehydrogenase. Biochemistry.

[19] Cho Y, Sharma V, Sacchettini JC (2003). Crystal structure of ATP phosphoribosyltransferase from Mycobacterium tuberculosis. J. Biol. Chem..

[20] Mittelstadt G, Moggre GJ, Panjikar S, Nazmi AR, Parker EJ (2016). Campylobacter jejuni adenosine triphosphate phosphoribosyltransferase is an active hexamer which is allosterically controlled by the twisting of a regulatory tail. Protein Sci..

[21] Tsai CJ, Nussinov R (2014). A unified view of “how allostery works”. PLoS Comput. Biol..

[22] Changeux JP (2012). Allostery and the Monod-Wyman-Changeux model after 50 years. Ann. Rev. Biophys.

[23] Botts J, Morales M (1953). Analytical description of the effects of modifiers and of enzyme multivalency upon the steady state catalyzed reaction rate. Trans. Faraday Soc..

[24] Sebastian JF (1987). Reversible activators of enzymes. J. Chem. Educ..

[25] Segel, I. H. *Enzyme Kinetics: Behavior and Analysis of Rapid Equilibrium and Steady-State Enzyme Systems* (Wiley, 1993).

[26] Andi B, West AH, Cook PF (2005). Regulatory mechanism of histidine-tagged homocitrate synthase from Saccharomyces cerevisiae. I. Kinetic studies. J. Biol. Chem..

[27] Wang YS, Liu D, Wyss DF (2004). Competition STD NMR for the detection of high-affinity ligands and NMR-based screening. Magn. Reson. Chem..

[28] Lohkamp B, McDermott G, Campbell SA, Coggins JR, Lapthorn AJ (2004). The structure of Escherichia coli ATP-phosphoribosyltransferase: identification of substrate binding sites and mode of AMP inhibition. J. Mol. Biol..

[29] Cho Y, Ioerger TR, Sacchettini JC (2008). Discovery of novel nitrobenzothiazole inhibitors for Mycobacterium tuberculosis ATP phosphoribosyl transferase (HisG) through virtual screening. J. Med. Chem..

[30] Rani C (2015). High-throughput screen identifies small molecule inhibitors targeting acetyltransferase activity of Mycobacterium tuberculosis GlmU. Tuberculosis.

[31] Wolan DW, Zorn JA, Gray DC, Wells JA (2009). Small-molecule activators of a proenzyme. Science.

[32] Clark MA (2009). Design, synthesis and selection of DNA-encoded small-molecule libraries. Nat. Chem. Biol..

[33] Jahnke W (2000). Second-Site NMR Screening with a Spin-Labeled First Ligand. J. Am. Chem. Soc..

[34] Sun Q (2014). A method for the second-site screening of K-Ras in the presence of a covalently attached first-site ligand. J. Biomol. NMR..

[35] Zorn JA, Wells JA (2010). Turning enzymes ON with small molecules. Nat. Chem. Biol..

[36] Milne JC (2007). Small molecule activators of SIRT1 as therapeutics for the treatment of type 2 diabetes. Nature..

[37] Pfefferkorn JA (2011). Designing glucokinase activators with reduced hypoglycemia risk: discovery of N,N-dimethyl-5-(2-methyl-6-((5-methylpyrazin-2-yl)-carbamoyl)benzofuran-4-yloxy)pyrimidine-2-carboxamide as a clinical candidate for the treatment of type 2 diabetes mellitus. Med. Chem. Commun..

[38] Wisastra R (2013). Discovery of a novel activator of 5-lipoxygenase from an anacardic acid derived compound collection. Bioorg. Med. Chem..

[39] Darby JF (2014). Discovery of selective small-molecule activators of a bacterial glycoside hydrolase. Angew. Chem..

[40] Leung E (2011). Activators of cylindrical proteases as antimicrobials: identification and development of small molecule activators of ClpP protease. Chem. Biol..

[41] Gersch M (2015). AAA + chaperones and acyldepsipeptides activate the ClpP protease via conformational control. Nat. Commun..

[42] Rashid Y, Kamran Azim M, Saify ZS, Khan KM, Khan R (2012). Small molecule activators of proteasome-related HslV peptidase. Bioorg. Med. Chem. Lett..

[43] Mayer M, Meyer B (1999). Characterization of Ligand Binding by Saturation Transfer Difference NMR Spectroscopy. Angew. Chem. Int. Ed..

[44] Hwang TL, Shaka AJ (1995). Water Suppression That Works. Excitation Sculpting Using Arbitrary Wave-Forms and Pulsed-Field Gradients. J. Magn. Reson. A.

[45] Winter G (2010). xia2: an expert system for macromolecular crystallography data reduction. J. Appl. Crystallogr..

[46] Kabsch W. (2010). Xds. Acta. Crystallogr. D. Biol. Crystallogr..

[47] The CCP4 suite: programs for protein crystallography (1994). Acta. Crystallogr. D Biol. Crystallogr..

[48] Hunter RW (2014). Mechanism of action of compound-13: an alpha1-selective small molecule activator of AMPK. Chem. Biol..

[49] Mujica-Jimenez C, Castellanos-Martinez A, Munoz-Clares RA (1998). Studies of the allosteric properties of maize leaf phosphoenolpyruvate carboxylase with the phosphoenolpyruvate analog phosphomycin as activator. Biochim. Biophys. Acta.

[50] Andi B, Cook PF (2005). Regulatory mechanism of histidine-tagged homocitrate synthase from Saccharomyces cerevisiae. II. Theory. J. Biol. Chem..

