# Supplementary material for: Uncoupling conformational states from activity in an allosteric enzyme
Source: Nat Commun. 2017 Aug 7;8:203. doi: 10.1038/s41467-017-00224-0 (PMC5545217; doi:10.1038/s41467-017-00224-0)
Supplement: Supplementary file 1 — Supplementary Information [file 41467_2017_224_MOESM1_ESM.pdf]

File Name: Supplementary Information
Description: Supplementary Figures, Supplementary Table, Supplementary Methods, Supplementary Reference.

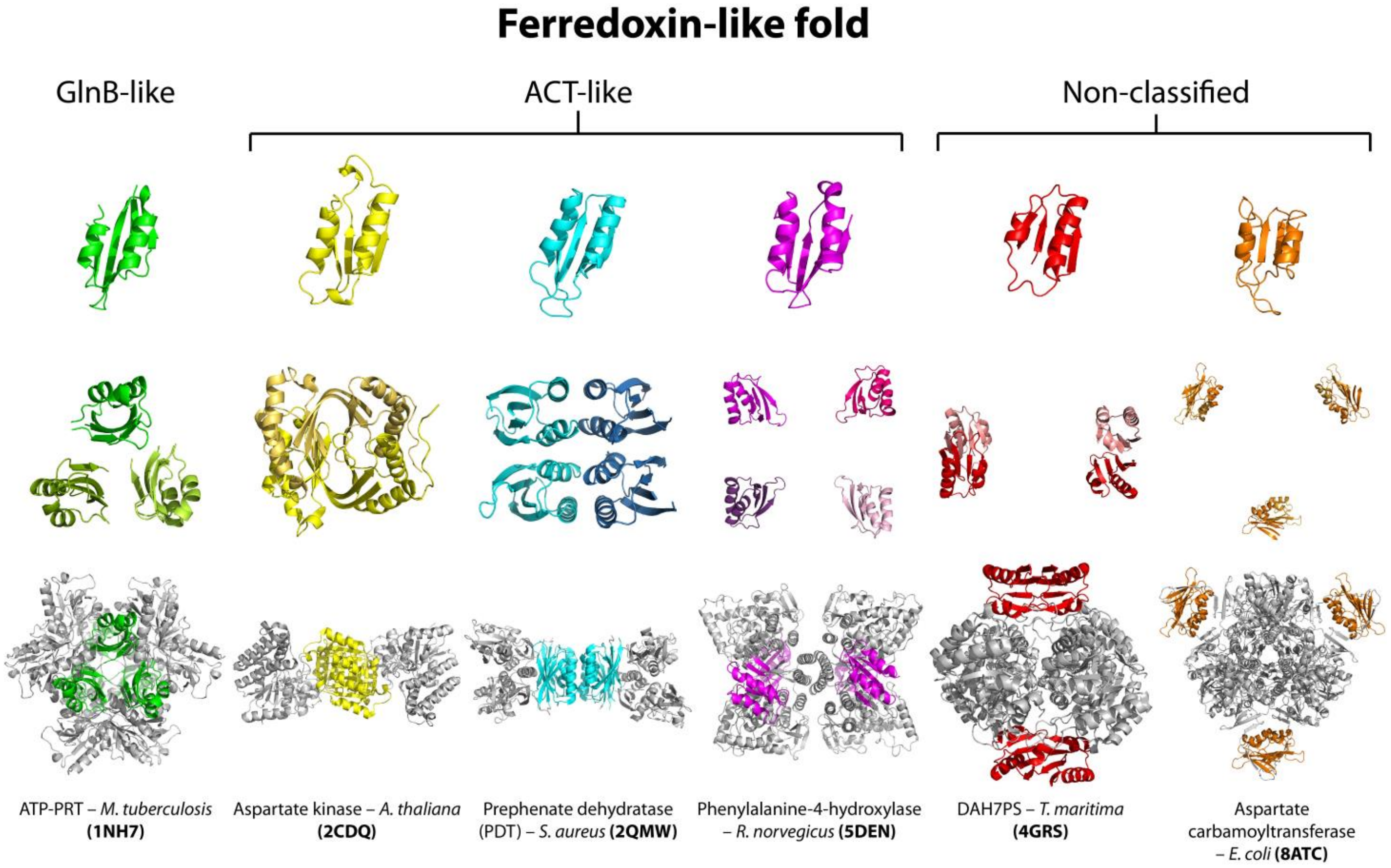

Supplementary Figure 1. Ferredoxin-like fold present in metabolic enzymes. Ferredoxin-like (FL) fold, here represented in 6 different enzymes from different superfamilies. All these enzymes are allosteric regulated by small-molecules. FL domains are coloured differently in each structure while catalytic domains are always coloured in grey. Top structures show the individual FL domains; middle structures show the relative arrangement between FL domains in the quaternary structure; and bottom structures are the full length oligomeric structures, alongside with their names, organism of origin and PDB code (in parentheses).

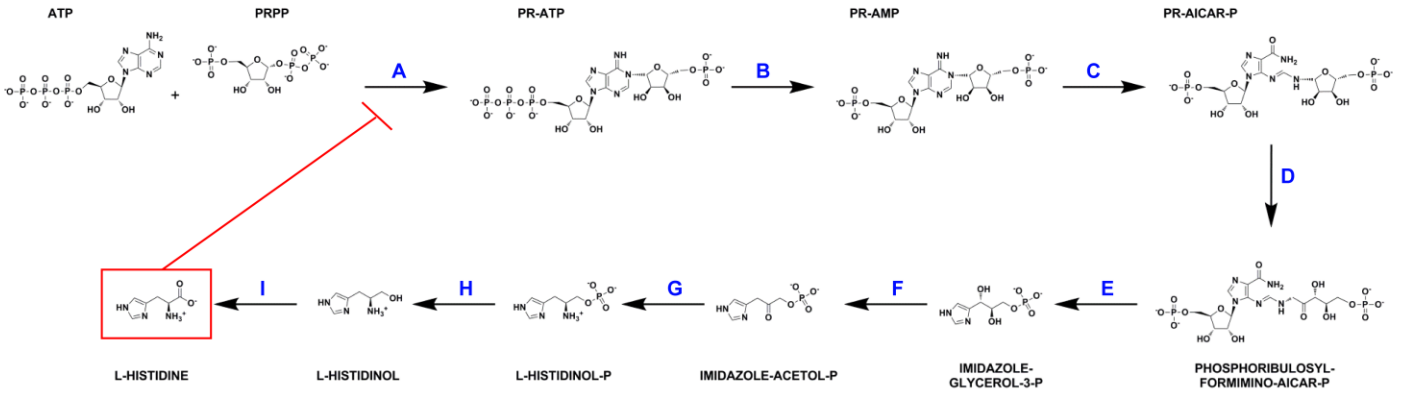

Supplementary Figure 2. L-His biosynthesis pathway. L-His, the end product of the pathway, allosterically feedback inhibits the first and committed step of the pathway, catalysed by ATP-PRT, directly regulating its own levels [1]. The enzymes involved in the pathway are:

A – ATP-phosphoribosyltransferase

B – Phosphoribosyl-ATP pyrophosphohydrolase

C – Phosphoribosyl-AMP cyclohydrolase

D – Phosphoribosyl isomerase A

E – Imidazole glycerol phosphate synthase

F – Imidazoleglycerol-phosphate dehydratase

G – Histidinol-phosphate aminotransferase

H – Histidinol-phosphate phosphatase

I - Histidinol dehydrogenase

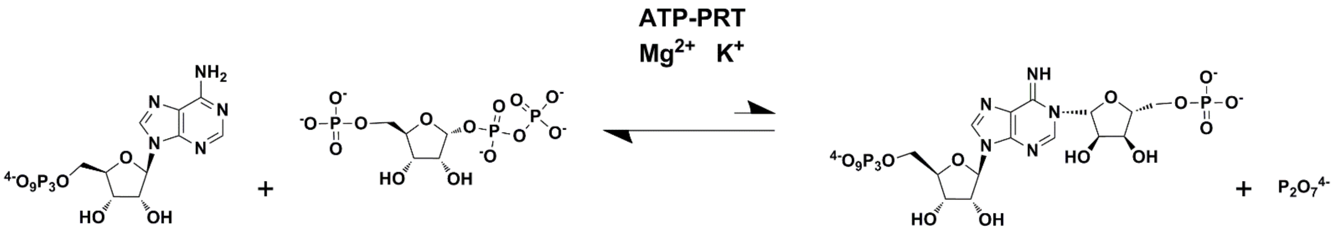

Supplementary Figure 3. ATP-PRT reaction. ATP-PRT catalyses the first and committed step in the histidine biosynthetic pathway, the $C_{1''}$-N_1 bond formation between ATP and 5-phospho-α-D-ribose 1-diphosphate, generating 1-(5-phospho-D-ribosyl)-ATP (PR-ATP) and inorganic pyrophosphate (PP_i). The reaction is Mg^{2+}-dependent, further activated by K^+ and readily reversible.

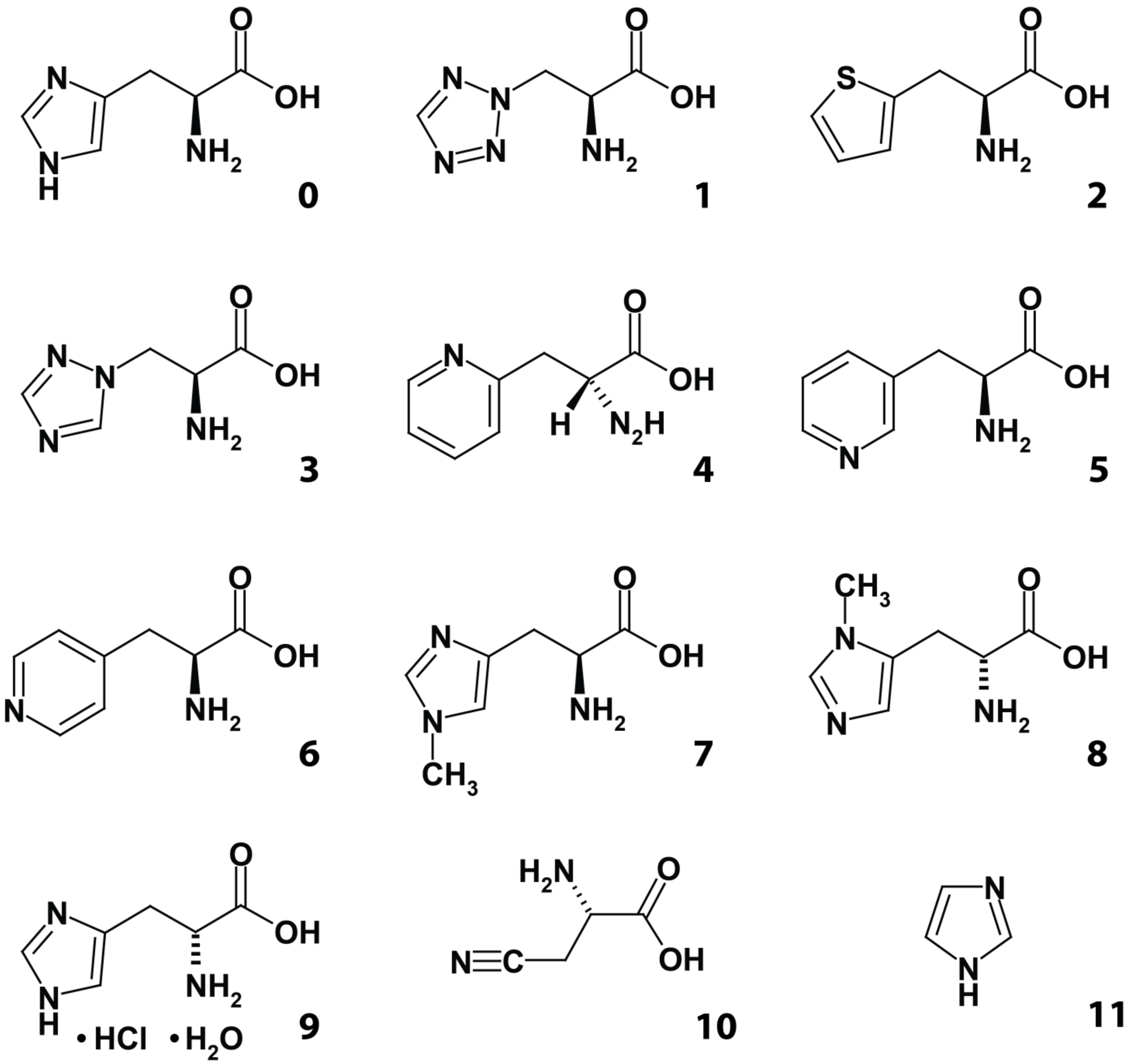

Supplementary Figure 4. L-His analogues used in CoSPI. (0) L-His; **(1)** 3-(2-Tetrazolyl)-L-alanine; **(2)** 3-(2-Thienyl)-L-alanine; **(3)** 3-(1,2,4-Triazol-1-yl)-L-alanine; **(4)** 3-(2-pyridyl)-L-alanine; **(5)** 3-(3-pyridyl)-L-alanine; **(6)** 3-(4-pyridyl)-L-alanine; **(7)** 1-Methyl-L-His; **(8)** 3-Methyl-L-His; **(9)** D-histidine monohydrochloride monohydrate; **(10)** β-Cyano-L-alanine; **(11)** Imidazole.

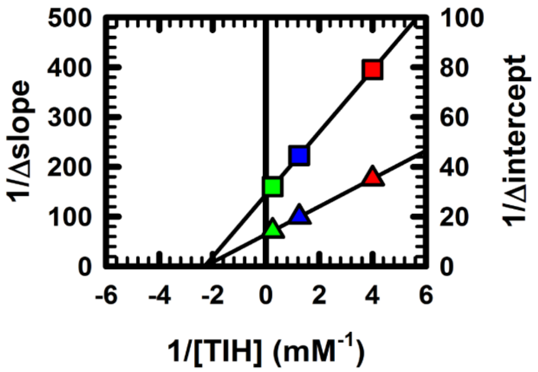

Supplementary Figure 5. Replot of the 1/Δintercept and 1/Δslope. Reploted from data shown in Figure 1F. 1/Δintercept (triangles) and 1/Δslope (squares). Points are data and the lines are linear regressions of the data.

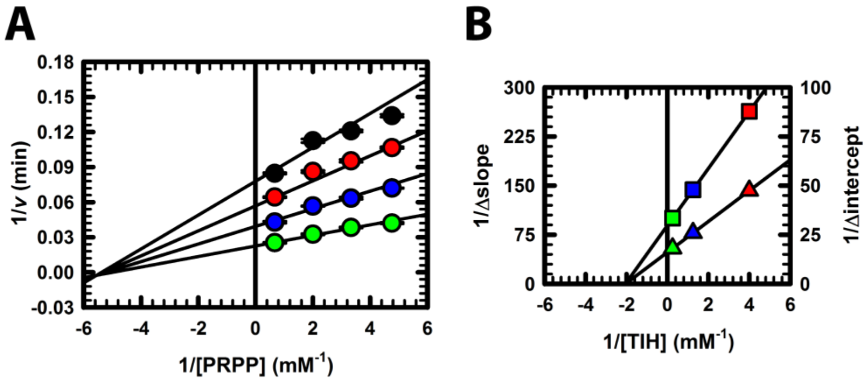

Supplementary Figure 6. Non-essential activation of ATP-PRT varying PRPP. (A) Double-reciprocal plot illustrating the linear, non-essential activation pattern obtained when varying the concentration of TIH at fixed variable concentrations of PRPP. Points are data obtained with 0 (black circles), 0.25 (red circles), 0.8 (blue circles) and 4 mM TIH (green circles), and error bars indicate the standard deviation (SD). Lines are the best fit of the entire data set to eq. 4. **(B)** Replot of the 1/Δintercept (triangles) and 1/Δslope (squares) of the data shown in panel *A*. Points are data and the lines are linear regressions of the data.

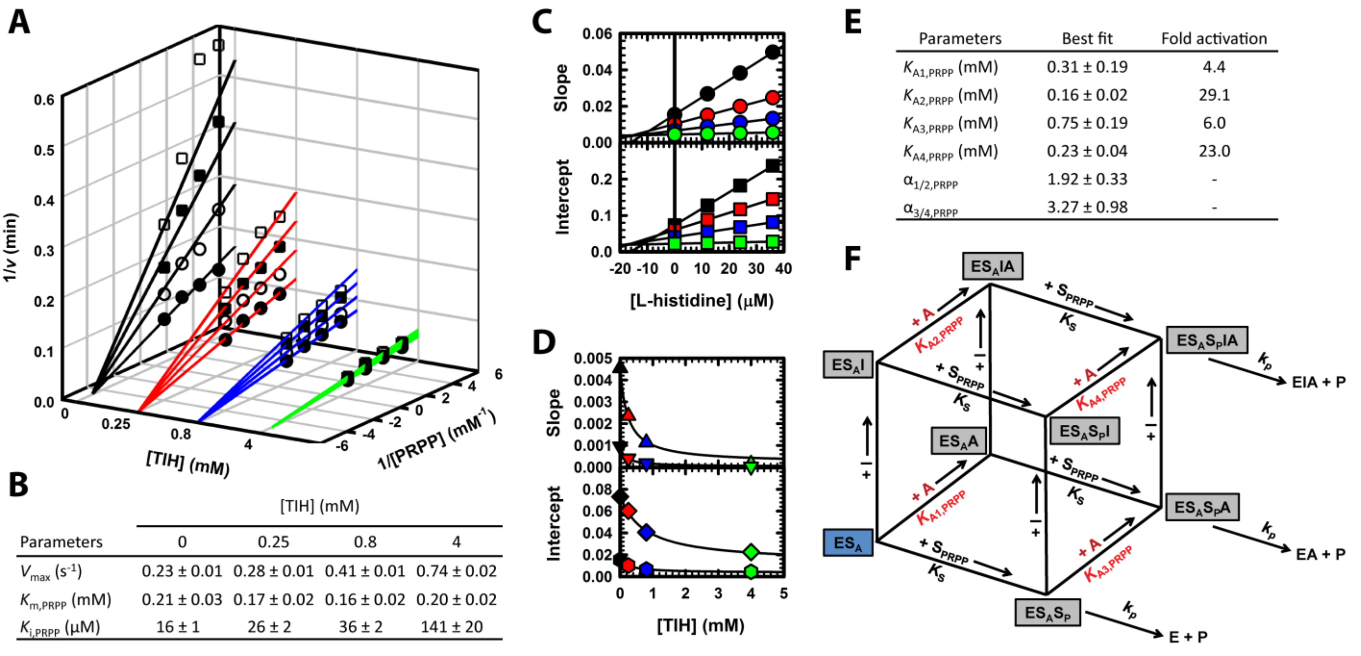

B

Parameters	[TIH] (mM) 0	0.25	0.8	4
V_{max} (s^{-1})	0.23 ± 0.01	0.28 ± 0.01	0.41 ± 0.01	0.74 ± 0.02
$K_{m,PRPP}$ (mM)	0.21 ± 0.03	0.17 ± 0.02	0.16 ± 0.02	0.20 ± 0.02
$K_{i,PRPP}$ (μM)	16 ± 1	26 ± 2	36 ± 2	141 ± 20

E

Parameters	Best fit	Fold activation
$K_{A1,PRPP}$ (mM)	0.31 ± 0.19	4.4
$K_{A2,PRPP}$ (mM)	0.16 ± 0.02	29.1
$K_{A3,PRPP}$ (mM)	0.75 ± 0.19	6.0
$K_{A4,PRPP}$ (mM)	0.23 ± 0.04	23.0
$\alpha_{1/2,PRPP}$	1.92 ± 0.33	-
$\alpha_{3/4,PRPP}$	3.27 ± 0.98	-

Supplementary Figure 7. L-His inhibition kinetics in the presence of TIH. L-His inhibition kinetics at fixed variable concentrations of PRPP and in the presence of TIH-induced activation. **(A)** Four double-reciprocal plots showing linear, noncompetitive inhibition patterns determined for L-His in the presence of 0 (black line), 0.25 (red line), 0.8 (blue line**)** and 4 mM (green line) of TIH, at fixed variable concentrations of PRPP. Results are representative of two independent experiments. Points are data obtained with 0 (close circles), 12 (open circles), 24 (close squares) and 36 µM L-His (open squares). Lines are the best fit of each noncompetitive pattern data set to eq. 6. **(B)** Steady-state kinetic parameters for ATP-PRT inhibition in the presence of TIH. **(C)** Secondary replot of the slopes (top panel) and intercepts (lower panel) of the data shown in panel *A*, illustrating the linear dependence on inhibitor concentration. Points are data, and the lines are linear regressions of the data. **(D)** Tertiary replots of the slopes (top panel) and intercepts (lower panel) of the data shown in panel C, illustrating an exponential dependence on activator concentration. Points are data and represent the slope of the intercept (triangles), slope of the slope (inverted triangles), intercept of the intercept (diamonds) and intercept of the slope (hexagons), and lines are the best fit to eq. 7. **(E)** Steady-state kinetic parameters for ATP-PRT activation in the presence of L-His. **(F)** Kinetic model for a combination of noncompetitive inhibition and non-essential activation. All the steps inside the cube are reversible. The blue box represents the enzyme complex always present in the assay.

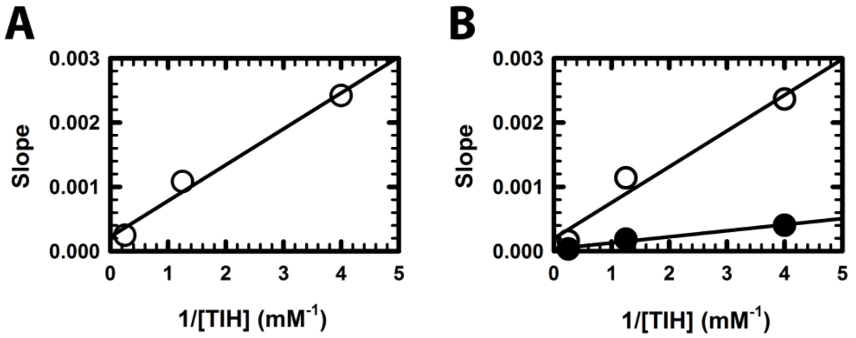

Supplementary Figure 8. Replot of the slopes. (A) Slope of the intercept from the experiment varying the substrate ATP. **(B)** Slope of the slope (closed circles) and slope of the intercept (open circles) from the experiment varying the substrate PRPP. Data from Fig. 3c and Supplementary Fig. 7d.

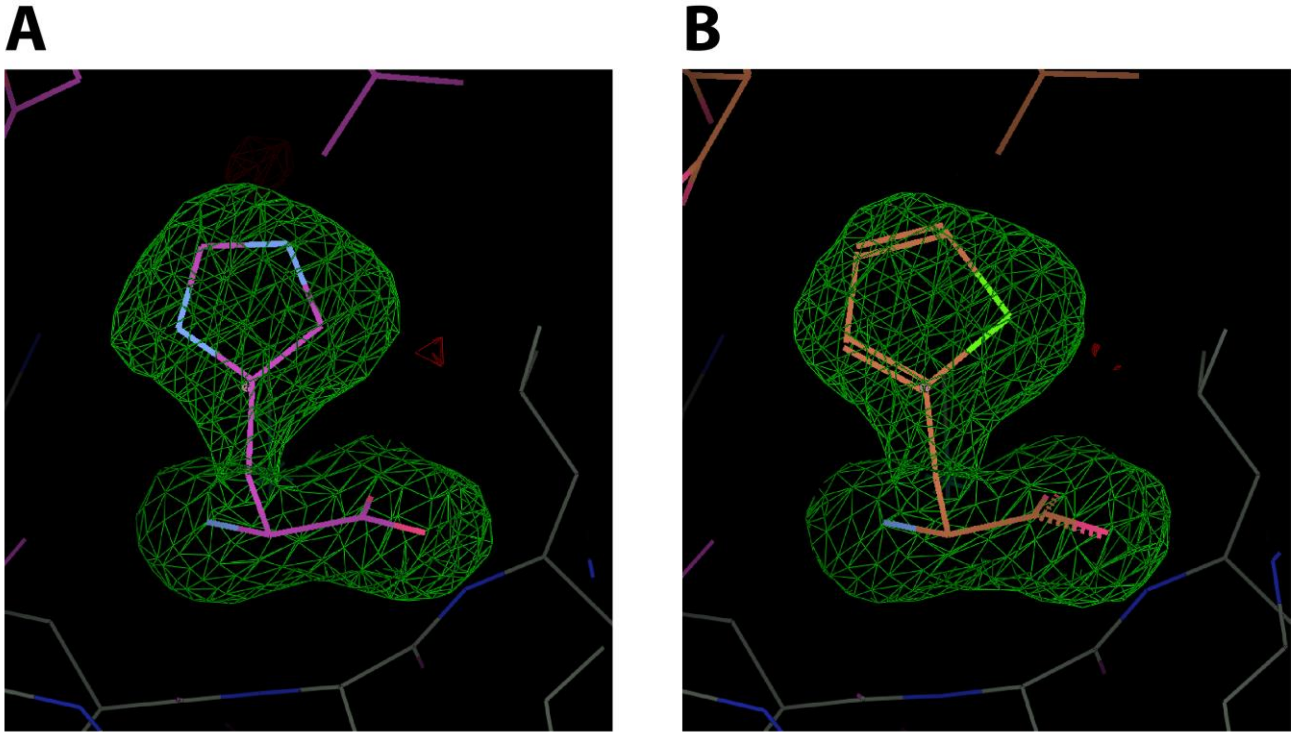

Supplementary Figure 9. Fo-Fc electron density map of the ligands. L-His **(A)** and TIH **(B)**. The map was computed after removal of ligand coordinates from the refined structures and contoured at 4σ. The ligand molecules from refined structures are shown. “Hardware stereo view”.

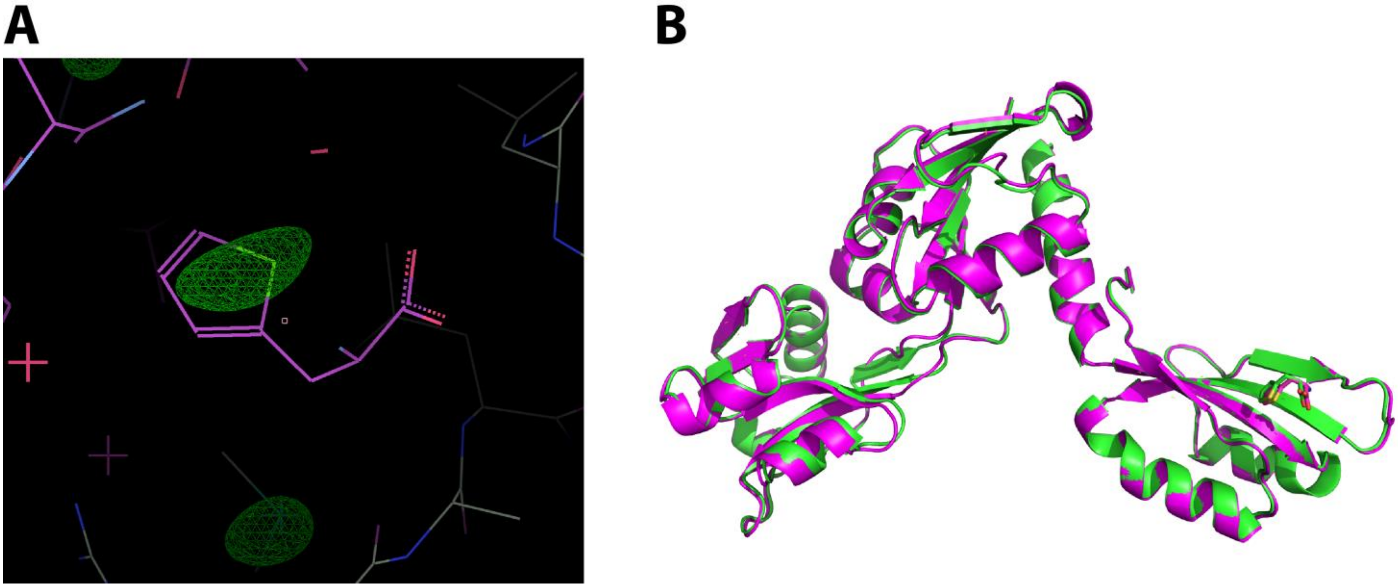

Supplementary Figure 10. Structure of ATP-PRT with TIH. (A) Anomalous scattering difference map (contoured at 4σ) calculated for a crystallographic dataset of the ATP-PRT/TIH complex at 1.76 Å (crystallized from condition 71 of the Protein Complex Suite by Qiagen: 0.1 M Sodium acetate anhydrous pH 5.0, 1.5 M Ammonium sulphate). Positive electron density marked the position of sulphur atoms in the ligand, as well as in surrounding methionine and cysteine side chains, and confirmed the identity of TIH. "Hardware stereo view". **(B)** Superposition of the ATP-PRT/TIH complex structure (monomer) obtained at 1.76 Å (**A**) (magenta) and the ATP-PRT/TIH complex (PDB code 5LHT) (green) obtained at 2.06 Å (crystallized from 4 of Classics Lite Suite crystallization screen by Qiagen: 2.5 %(v/v) Isopropanol, 1.0 M Ammonium sulphate). Crystallographic statistics are reported in Table S1.

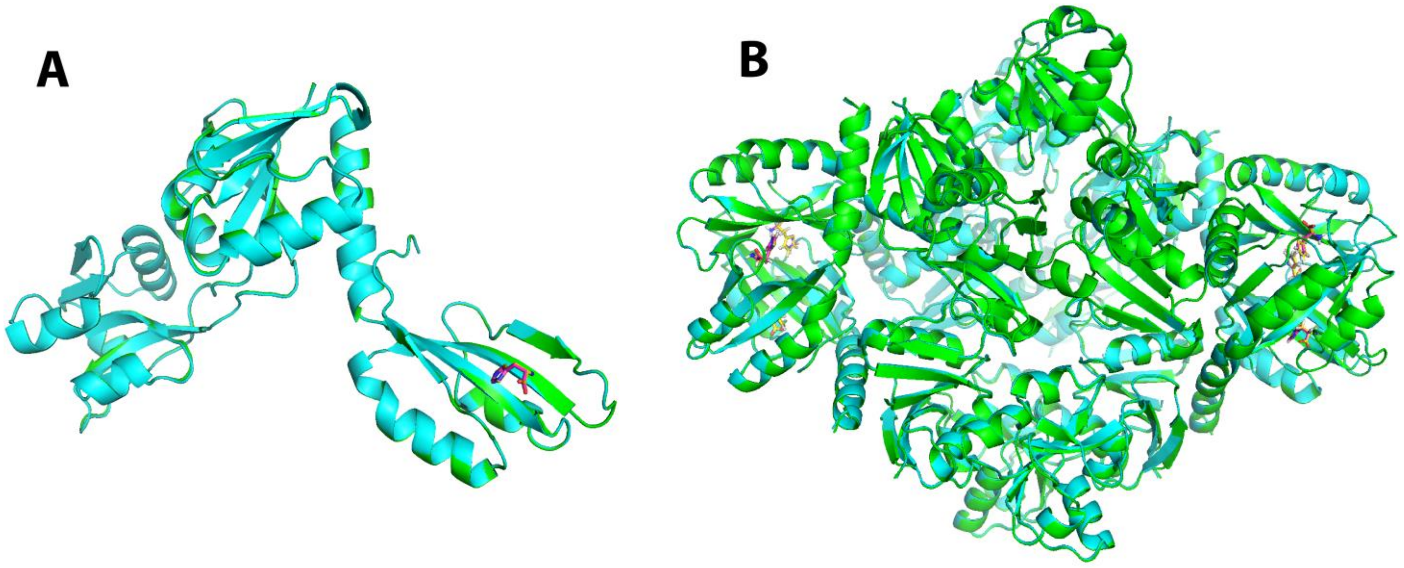

Supplementary Figure 11. Superposition of ATP-PRT in complex with L-His or TIH. Superposition of the ATP-PRT/L-His (cyan) and ATP-PRT/TIH (green) complexes (this study) as crystallographic hexamers obtained by symmetry operation. L-His and TIH are in magenta and yellow, respectively.

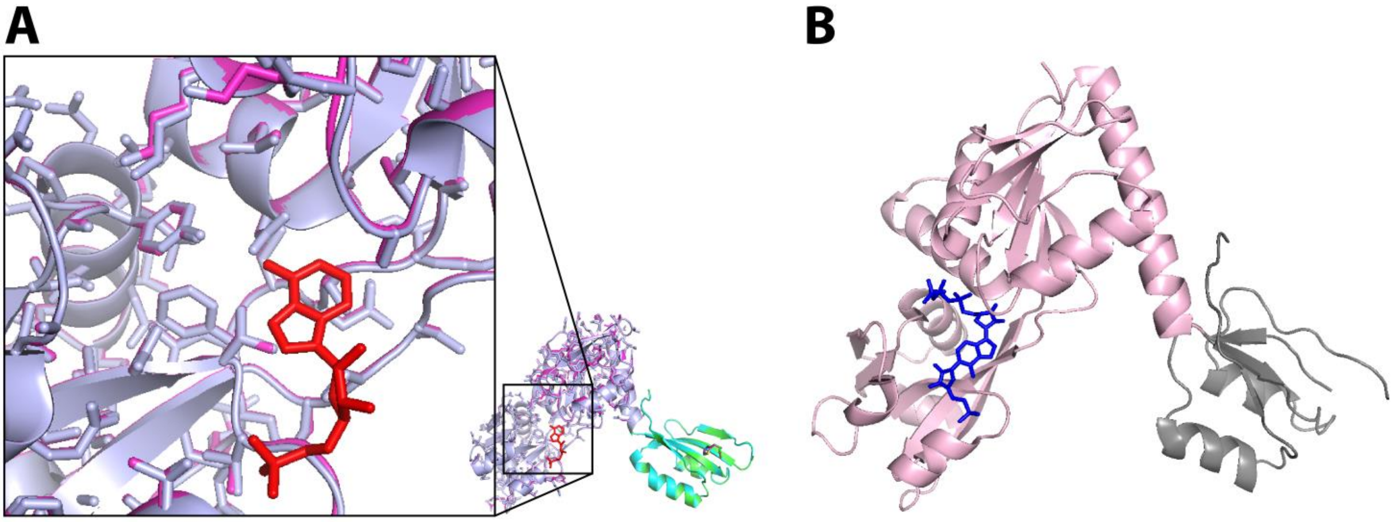

Supplementary Figure 12. Active site of ATP-PRTs. (A) Superposition of the ATP-PRT/L-His and ATP-PRT/TIH complexes (this study) where allosteric and catalytic domain are shown in cyan/light blue and green/magenta, respectively for the two structures. The zoomed section of the catalytic domains highlights the perfect superposition of the side-chains expected to be involved in the substrate/product binding. To help localizing the active-site, the coordinates of AMP from 1NH8 (binding in the putative PRPP site), have been included after superposing the protein coordinates. **(B)** *E.coli* ATP-PRT in complex with product PR-ATP (1Q1K) in blue oriented as Mtb ATP-PRT in (**A**).

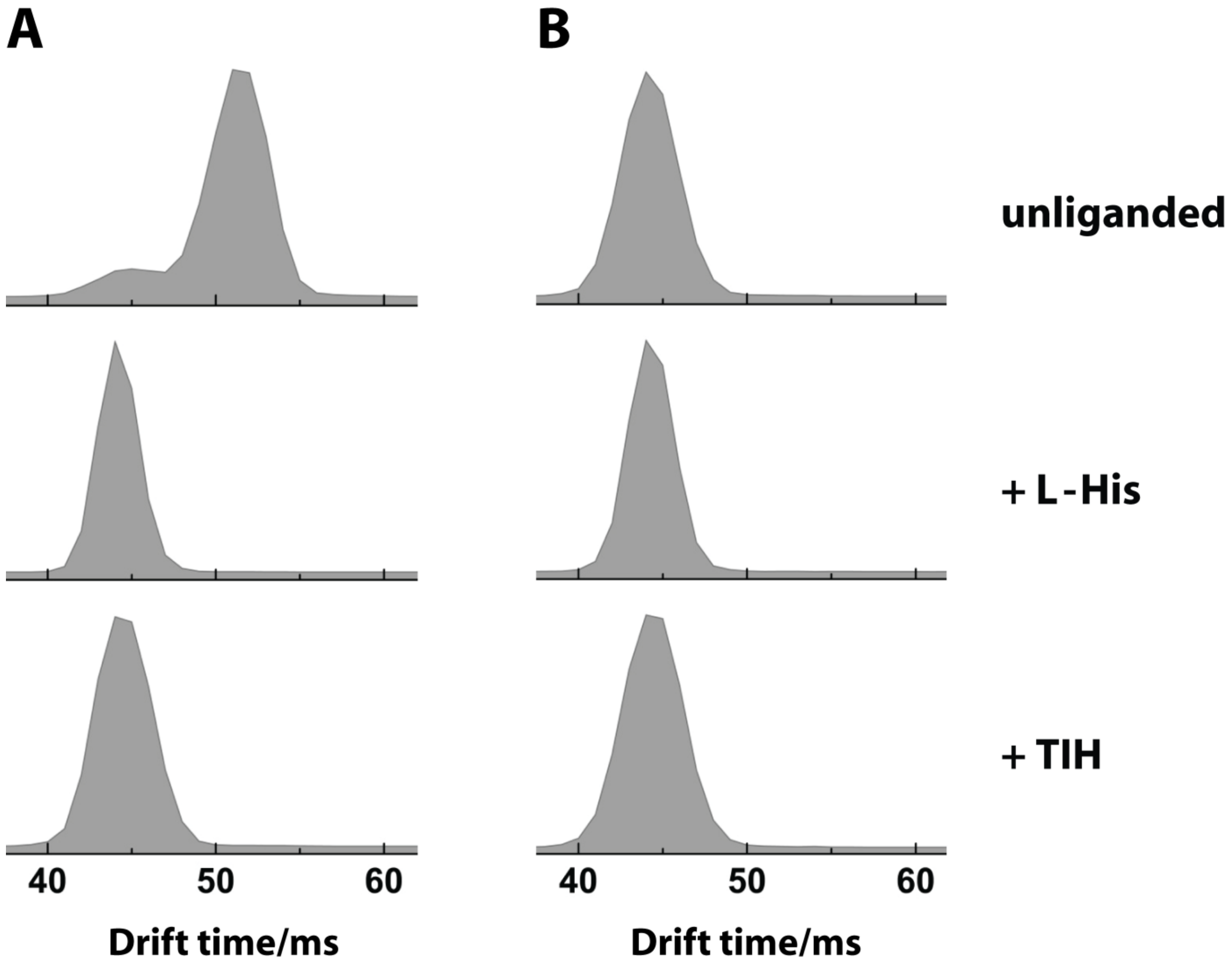

Supplementary Figure 13. Ion mobility MS data. Arrival time distributions of ATP-PRT at neutral pH (6.8) (A) and basic pH (9.0) (B).

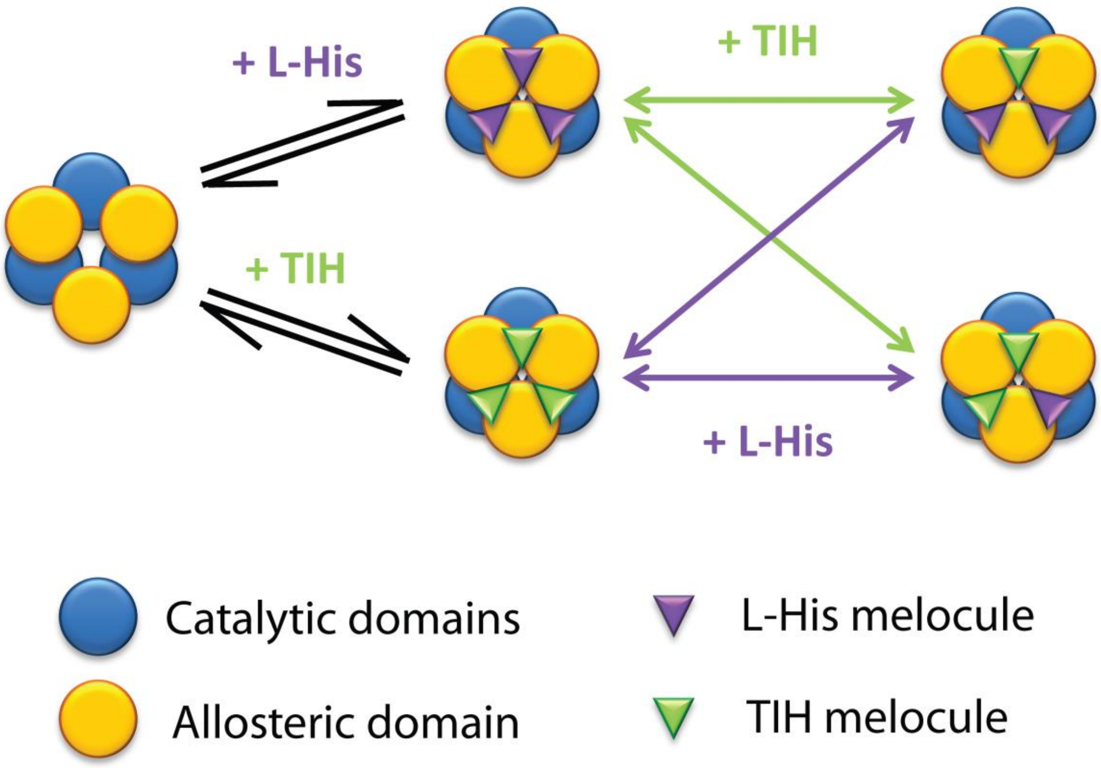

Supplementary Figure 14. Co-occupancy of the allosteric site by L-His and TIH. This scheme shows a view from the top of ATP-PRT where it is possible to observe 3 allosteric domains. The other 3 exact same allosteric domains from the hexamer are placed on the opposite side, not visible.

Supplementary Table 1. Drift times for main ATP-PRT form at different pH and in the presence of L-His and TIH

Charge state	Ligand	pH 6.8	pH 9.0
	None	51.2 ms	44.0 ms
**(+28)**	L-His	44.1 ms	44.2 ms
	TIH	44.2 ms	44.1 ms

Supplementary methods

Determination of the magnitude of activation by TIH

The fold activation by TIH is calculated from the fit of the data of the tertiary replots with covariation of ATP (Fig. 3C) and PRPP (Supplementary Fig. 3D) to equation 7. The fold activation is given by y_0/y_∞, where y_0 is the y value in the absence of TIH and y_∞ is the y value in the presence of infinite concentration of TIH. This calculation was straightforward for the ‘intercepts of the slopes’, binding of TIH to ES_P and ES_A, with ratios of 10.9 and 4.4, respectively, and also for the ‘intercepts of the intercepts’, binding of TIH to ES_PS_A and ES_AS_P, with ratios of 6.6 and 6.0, respectively. However, due to experimental problems, the values of y_∞ for ‘slope of the slope’, binding of TIH to ES_AI, and ‘slopes of the intercepts’, binding of TIH to ES_PS_AI and ES_AS_PI, were close to zero at infinite TIH. Therefore, the values had to be obtained by fitting the slopes versus the reciprocal of TIH concentration (Suppementary Fig. 8). Positive y_∞ values, corresponding to the y-interception of the linear regressions were obtained. The data in Fig. 3C and Supplementary Fig. 7D, were then fitted with equation 7 fixing the y_∞ values obtained from the reciprocal plots. Finally, the fold activation values were calculated, 29.1-fold for ‘slope of the slope’ and 18.1- and 23.0-fold for ‘slope of the intercept’ when varying ATP and PRPP, respectively.

Supplementary References

1. Ames, B.N., Martin, R.G. & Garry, B.J. The first step of histidine biosynthesis. *J Biol Chem* **236**, 2019-26 (1961).
